# Monoamine Oxidase‐B (MAO‐B) Identifies Neurotoxic Reactive Astrocytes in Alzheimer's Disease and Related Dementias

**DOI:** 10.1002/alz70855_102019

**Published:** 2025-12-23

**Authors:** Methasit Jaisa‐aad, Cinthya Aguero, Neus Rabaneda‐Lombarte, Celia R. Bianco, Alexandra N. Melloni, Bradley T. Hyman, Teresa Gomez‐Isla, Pedro Brugarolas, Alberto Serrano‐Pozo

**Affiliations:** ^1^ MassGeneral Institute for Neurodegenerative Disease, Charlestown, MA, USA; ^2^ Harvard Medical School, Boston, MA, USA; ^3^ Massachusetts General Hospital, Boston, MA, USA; ^4^ Massachusetts Alzheimer's Disease Research Center, Charlestown, MA, USA

## Abstract

**Background:**

Single nucleus RNA‐sequencing has revealed the complexity and heterogeneity of reactive astrogliosis in Alzheimer's disease (AD) and AD‐related dementias (ADRD). Our recent large snRNA‐seq study comprising 5 brain regions and 32 brain donors representing the stereotypical hierarchical spatio‐temporal progression of AD identified new markers of homeostatic and reactive astrocytes as well as subclusters with specialized functions, transcriptomic changes along AD‐vulnerable neural networks and in parallel to Aβ plaque and pTau accrual, and potential signatures of regional vulnerability and resilience in the normal aging brain, overall supporting the idea that astrocytes contribute to the selective neuronal vulnerability that characterizes AD/ADRD. Here we focused on evaluating monoamine oxidase‐B (MAO‐B) as a reactive astrocyte marker in AD/ADRD given its implication in oxidative stress and GABA tonic inhibition and that MAO‐B radioligands have been proposed for PET imaging of reactive astrogliosis.

**Method:**

Quantitative immunohistochemical study of MAO‐B, Aβ, pTau, pTDP43, GFAP, and YKL‐40 in formalin‐fixed paraffin‐embedded sections from the frontal association cortex (BA8/9) of donors with no primary neuropathological diagnosis (controls, *n* = 20), AD (*n* = 30), Pick's disease (PiD, *n* = 20), progressive supranuclear palsy (PSP, *n* = 20), cortico‐basal degeneration (CBD, *n* = 19), and frontotemporal lobar degeneration‐TDP (FTLD‐TDP, *n* = 20). Cortical thickness was also measured as a proxy for severity of atrophy. The cell type(s) expressing MAO‐B was assessed with confocal microscopy and subcellular localization was investigated with super‐resolution microscopy.

**Result:**

Compared to controls, MAO‐B immunoreactive area fraction was significantly increased in the frontal cortex and white matter of AD, CBD, and especially PiD and FTLD‐TDP, but not PSP. MAO‐B area fraction correlated positively with that of GFAP and YKL‐40. MAO‐B was primarily expressed by cortical astrocytes across AD/ADRD and, as expected, colocalized with their mitochondria. As reported for other reactive markers, tufted astrocytes in PSP tended to have low MAO‐B levels. Importantly, multivariable linear regression analysis revealed a significant negative association between MAO‐B area fraction and cortical thickness even after adjusting for Aβ, pTau or pTDP43 burdens.

**Conclusion:**

MAO‐B up‐regulation may identify the subset of reactive neurotoxic astrocytes in AD/ADRD. Multimodal PET‐MRI studies are needed to test the hypothesis that MAO‐B radioligand binding predicts future neurodegeneration in individuals with AD/ADRD.

